# Principle component analysis of myocardial strain to quantify left ventricular dyssynchrony

**DOI:** 10.1186/1532-429X-15-S1-P74

**Published:** 2013-01-30

**Authors:** Raghav Ramachandran, Xiao Chen, Bhairav B Mehta, Kenneth C Bilchick, Frederick H Epstein

**Affiliations:** 1 Biomedical Engineering, University of Virginia, Charlottesville, VA, USA; 2 Department of Medicine, University of Virginia, Charlottesville, VA, USA

## Background

Cardiac resynchronization therapy (CRT) is effective for selected heart failure (HF) patients, but is associated with a 30-40% nonresponse rate. Identification of CRT responders may be improved with myocardial strain imaging. The circumferential uniformity ratio estimate (CURE )^1^ measures mechanical dyssynchrony by Fourier series fitting of myocardial strains over space, but requires user interaction to define a range of cardiac phases over which CURE is calculated (time dependence). We hypothesize that principal component analysis (PCA) can quantify dyssynchrony in myocardial strain in a data-driven, time-independent manner that does not require any subjective user assessments of strain data.

## Methods

Dyssynchronous HF was induced in canines (N=5) with tachycardia pacing and left bundle branch ablation (LBBB-HF), while synchronous HF with narrow QRS (NQRS-HF) was induced in canines with tachycardia pacing only (N=5). Four normal canines were also studied.

Spiral cine DENSE MRI was performed on a 1.5 T scanner (Avanto, Siemens) in all 14 canines. 2D myocardial motion was tracked in a mid-ventricular short-axis plane from DENSE images acquired using the following parameters^2^: interleaves=6, TR=17 ms, TE=1.9 ms, slice thickness=8 mm, excitation flip angle=15°, in-plane resolution=2.8 x2.8 mm and displacement-encoding frequency= 0.1 cycles/mm.

DENSE images were analyzed^3^ to calculate left ventricular (LV) circumferential strain (Ecc), and PCA was applied to the Ecc-time curves for a 24-segment LV model. Specifically, the LV Ecc curves were decomposed spatially into principal component basis vectors. The PCA-based metric for measuring LV dyssynchrony, termed First Order Regional Conformity Estimate (FORCE), was calculated as |sum(PCL_1_)|/sum(|PCL_1_|) where PCL_1_ represents the loadings of the first principal component basis vector. Both FORCE and CURE range from 0 (dyssynchrony) to 1 (perfect synchrony). The Kruskal-Wallis one-way ANOVA test was used to compare FORCE and CURE among the three groups in pairwise fashion.

## Results

**Figure 1:**
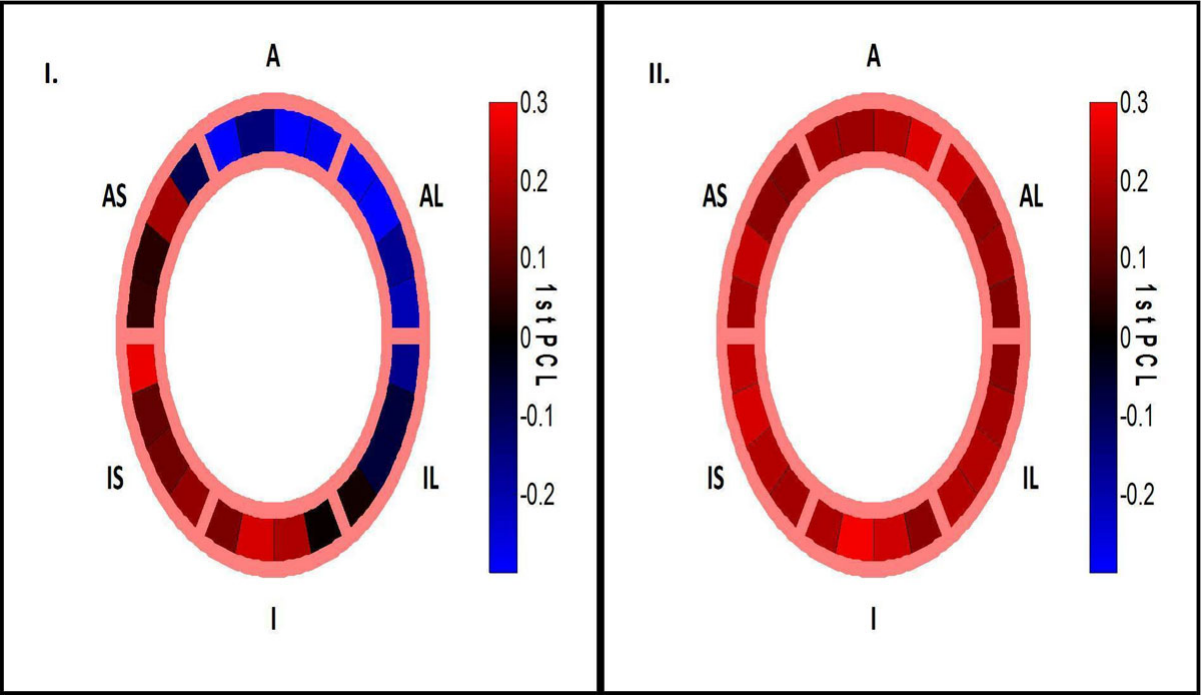
Bulls-eye plots of PCL_1_ as a function of LV segments for I) a LBBB-HF and II) a NQRS-HF canine; A-Anterior, AL-Anterolateral, IL-Inferolateral, I-Inferior, IS-Inferoseptal, AS-Anteroseptal

**Figure 2:**
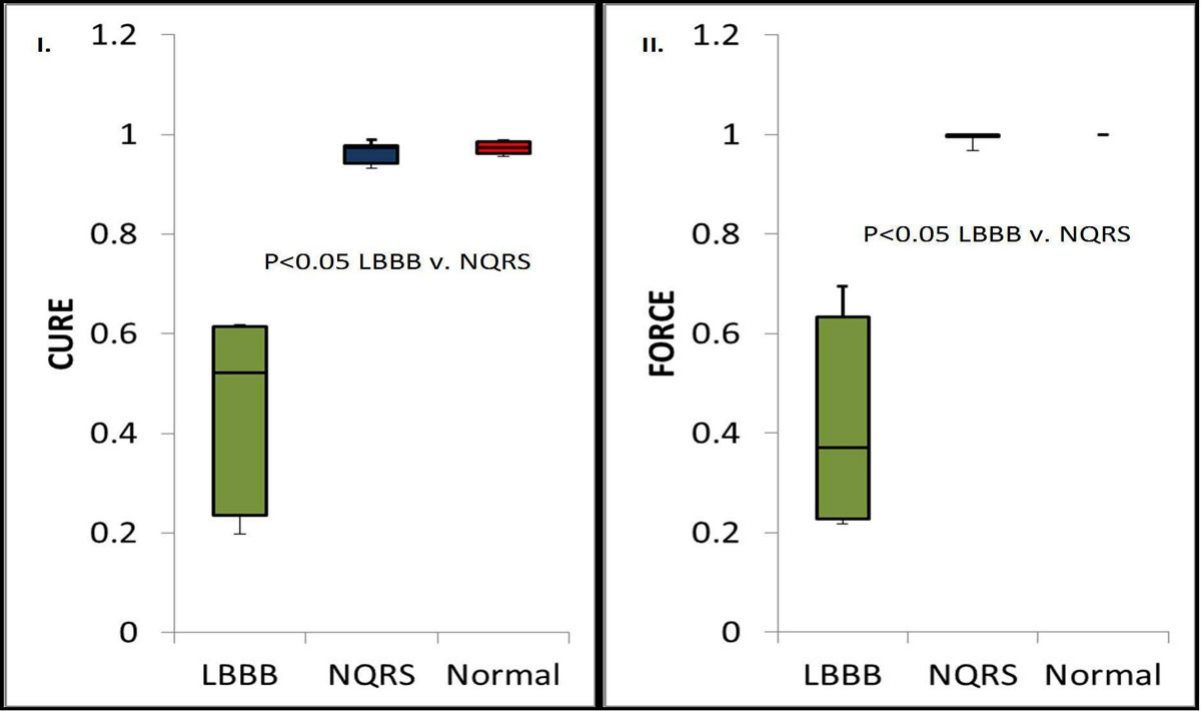
Boxplot comparisons of I) CURE and II) FORCE for LBBB-HF,NQRS-HF and Normal dogs

Figure 1 shows the spatial distribution of PCL_1_ for the Ecc of example LBBB-HF and NQRS-HF canines. The PCL_1_ of LV Ecc in LBBB-HF canines varied widely over LV segments, whereas PCL_1_ showed little variation over LV segments in NQRS-HF and normal canines. As shown in Figure 2, FORCE and CURE were both markedly different in LBBB-HF versus NQRS-HF (p<0.05) and LBBB-HF versus normal (p<0.05). Also, FORCE was significantly greater than CURE for NQRS-HF (p<0.05), indicating even better identification of synchrony than CURE.

## Conclusions

PCA, using the promising new FORCE parameter, effectively and automatically identifies mechanical dyssynchrony in HF in a data-driven and completely time-independent fashion. Further clinical evaluation of FORCE for prediction of CRT response is warranted.

## Funding

This work was funded by the American Heart Association 12GRNT12050301 and NIH Heart, Lung and Blood Institute (NHLBI) T32HL007284.
